# Evaluating the physicochemical properties of camelina (*Camelina sativa*) seed oil obtained through optimized ultrasonic-assisted extraction

**DOI:** 10.1016/j.ultsonch.2025.107371

**Published:** 2025-04-24

**Authors:** Samira Mansuri, Hamid Bakhshabadi, Masumeh Moghimi, Aminallah Tahmasebi, Mehdi Gharekhani

**Affiliations:** a Department of Food Science and Technology, GonbadKavoos Branch, Islamic Azad University, Gonbad-e-Kavoos, Iran; b Department of Agriculture, Minab Higher Education Center, University of Hormozgan, Bandar Abbas, Iran; c Department of Chemistry, GonbadKavoos Branch, Islamic Azad University, Gonbad-e- Kavoos, Iran; d Department of Food Science and Technology, Tabriz Branch, Islamic Azad University, Tabriz, Iran

**Keywords:** Camelina seeds, Fatty acid, Oxidative stability, Ultrasound waves

## Abstract

The primary limitation associated with oil extraction through pressing is the considerable amount of residual oil in the cake. Therefore, this study focused on employing ultrasound waves to decrease the oil content in camelina seeds. For this purpose, three ultrasound treatment durations (15 to 45 min) were applied, and oil was immediately extracted from the seeds using a screw press at varying speeds (11 to 55 rpm). Various physicochemical tests were conducted on the extracted oil. After identifying the optimal treatments, the fatty acid content and oxidative stability of the samples were determined. Results indicated that longer ultrasound treatment durations led to higher acidity and peroxide values in the samples. In contrast, the oil extraction efficiency and total phenol content initially increased and then declined. Increasing the rotational speed of the screw press decreased total phenol efficiency but increased acidity and peroxide values. Ultrasound pretreatment had no effect on the refractive index of the oils. Based on the process optimization results, ultrasound pretreatment achieved optimal oil extraction from camelina seeds at a treatment time of 21.02 min and a screw press speed of 11 rpm. Under these conditions, the extraction parameters included an efficiency of 34.5 %, an oil acidity of 0.394 % (as oleic acid), a peroxide value of 0.97 meqO_2_/kg oil, a total phenol content of 122.68 ppm, and a refractive index of 1.4750. Ultrasound pretreatment also reduced oxidative stability from 3.75 h to 3.13 h. Gas chromatography results showed that linolenic acid was the major fatty acid in both treated and control oil samples. As a result, the findings demonstrate that ultrasound pretreatment is an effective method for extracting oil from camelina seeds.

## 1 Introduction

The increased interest in cultivating oilseeds among farmers and governments stems from their various applications, including use in human food, animal feed, pharmaceuticals, soap-making, and as a source of fuel. With the rise in public awareness, there has been growing demand for oils that offer both energy and flavor while also having a beneficial impact on health. Considering that Iran depends significantly on imports for its vegetable oil requirements—over 94 % of the oil consumed in the country is sourced from abroad—leveraging domestic resources to achieve self-sufficiency, enhancing the cultivation of prevalent oilseeds, and exploring new sources are essential for meeting the nation’s oil requirements [1,2]. Camelina is a new oil source that has recently gained attention in the oil extraction industry. Camelina (Camelina sativa L.), belonging to the Brassicaceae family, is also known as false flax or German sesame. This plant is highly adaptable, growing in various soils and climates. Additionally, its cultivation is cost-effective, requiring less water, fertilizer, and pesticides compared to other oilseeds such as sunflower, soybean, or canola [3,4]. Historically, this plant was cultivated for biodiesel and cosmetic industries, but it has recently gained attention as a food product due to its bioactive and health-promoting compounds [3]. The camelina seed contains 30 % to 40 % oil by dry weight, and its oil is rich in polyunsaturated fatty acids, especially linoleic and linolenic acids, which make up more than 50 % of the oil's fatty acids. Moreover, this oil contains high amounts of tocopherols, which protect it against oxidation, making it suitable for use in various food products. Moreover, due to its high protein content, camelina seeds are also used in the animal feed industry [5].

One method of oil extraction is mechanical extraction (pressing), which is typically used on a small scale. Mechanical oil extraction methods are divided into two types: hot pressing and cold pressing. Hot pressing yields higher oil extraction efficiency than cold pressing; however, the heat generated during hot pressing reduces oil quality. In contrast, cold-pressed oil retains its natural properties and is free from chemicals. As a result, demand for cold-pressed oils is increasing [6]. In cold-press oil extraction, various factors such as press pressure, screw press rotation speed, seed moisture, and process temperature affect the extraction efficiency. In cold-press extraction, the absence of chemicals ensures that the oil and meal are free from chemical compounds, making their consumption suitable for both humans and animals. The main limitation of oil extraction by pressing is the high residual oil content in the meal, which can reach 10–20 % in some cases. During this process, the moisture content and temperature of the raw material must also be strictly controlled to prevent damage to the meal's proteins [7]. To solve this problem, the industry uses solvents to extract oil, which raises environmental concerns [8]. Therefore, researchers have recently focused on techniques such as ultrasound extraction [9], microwave extraction, and pulsed electric fields [10]. Ultrasound-assisted extraction is one of the green technologies widely used in various industries. This process is based on cavitation, which occurs through ultrasonic probes or baths operating at frequencies of 20 and 40 kHz, respectively [11]. In a liquid medium, sound waves produced by the device propagate through the environment, creating alternating cycles of compression (high pressure) and rarefaction (low pressure), which generate cavitation. This allows for the intense mixing of solvent and solid particles. The bubbles formed by cavitation result in the violent collision of particles, which increases both internal and vortex diffusion resulting in the breakdown of the plant cell wall and an increase in mass transfer and oil extraction rate, resulting in the breakdown of the plant cell wall and an increase in mass transfer and oil extraction rate [12,13]. Ultrasound can increase gas–liquid mass transfer by up to five fold and liquid–solid mass transfer by 20–25 folds. This method eliminates the limitation of poor mixing between reactants, emulsifies reactants, and reduces the amount of catalyst, reaction time, and temperature [14]. Ultrasound-assisted extraction prevents the destruction of plant extracts and lowers energy consumption when extracting bioactive compounds from plants [15]. Researchers such as Moghimi et al. [9], Saghali et al. [7], and Thilakarathna et al. [11] have applied these waves in oil extraction. This method offers advantages such as low temperature, time, and energy consumption, low solvent usage, compatibility with various solvents, wide applicability across different plants, enhanced quality of extracted material (e.g., antioxidant properties), and extraction of heat-sensitive compounds without significantly altering fatty acid levels in oils. Studies suggest that ultrasound-assisted extraction is a promising method for industrialization compared to other modern extraction techniques like microwave, ohmic, and pulsed electric field methods [8]. Dar et al. [16] found that ultrasound improves oil extraction efficiency compared to the supercritical fluid method, increasing bioactive compounds in extracted oil compared to the supercritical fluid and Soxhlet method. Given the limited research on ultrasound pretreatment for oil extraction from camelina seeds, this study aims to investigate its application in the extraction of oil from camelina seeds.

## 2 Materials and methods

### 2.1 Preparation of camelina seeds and oil extraction

In this study, *Camelina* seeds with 38.5 % oil content were obtained from Khorasan Cotton and Oilseeds Company and transferred to the Food Industry Laboratory at Islamic Azad University, Gonbad Kavous Branch. Foreign materials such as weed seeds, sand, and stones were manually removed. The seeds were treated withultrasonic waves in a bath (Agilent, USA) made of stainless steel (with a frequency of 40 kHz, intensity of 75 %, and power of 150 W) at a temperature of 20 °C for three different durations (15, 30, and 45 min) [9]. The oil was extracted using a screw press (Kern Kraft, Germany) with a capacity of 8 kg per hour at three rotational speeds: 11, 33, and 55 rpm [1].

### 2.2 Determining oil extraction efficiency

Oil extraction efficiency from camelina seeds was determined according to the method by Bakhshabadi et al. [1], using a digital scale (Gec Avery, UK) with an accuracy of 0.01 g, based on Equation (1).

(1) $$E=\frac{M}{W}\times 100$$

Where E is the oil extraction efficiency (%), M represents the weight of oil obtained (g), and W shows the weight of initial seeds.

### 2.3 Acidity measurement

The acidity of the oil was measured using the AOCS method [17]. Initially, 5 g of oil was mixed with 20–30 mL of ethanol or another neutral alcohol, and a few drops of phenolphthalein were added. The mixture was titrated with 0.1 N sodium hydroxide until a pink color appeared. The acid number was calculated using Equation (2).

(2) $$A=\frac{2.82\;\times V}{\;W}$$

Where V is thevolume of sodium hydroxide used (mL), W shows the weight of the sample (g), and A represents the free fatty acids as oleic acid per 100 g of the sample.

### 2.4 Peroxide value determination

Peroxide value was determined following the AOCS method [17]. Five grams of oil was weighed into a 250 mL Erlenmeyer flask, and 300 mL of an acetic acid-chloroform solution (3:2 ratio) was added. After mixing, 0.5 mL of saturated potassium iodide solution was added, and the mixture was stored in the dark for 1 min. Thirty milliliters of distilled water was then added, and the solution was titrated with 0.1 N sodium thiosulfate until the yellow color disappeared. After adding 0.5 mL of starch indicator solution, titration continued until the blue color disappeared, and the peroxide value was calculated using Equation (3).

(3) $$P=\frac{S\times M\times 100}{\;W}$$

Where S shows the volume of sodium thiosulfate used (mL), M is the molarity of sodium thiosulfate, W represents the weight of oil (g), and P is the peroxide value (meq O_2_ per kg of oil).

### 2.5 Refractive index measurement

The refractive index of the oil was measured using a refractometer (Atago, Japan) at 25 °C, following the AOCS method (1993).

### 2.6 Total phenolic content measurement

Total phenolic content was determined by a colorimetric method using the Folin-Ciocalteu reagent. One gram of each sample was mixed with 3 mL of methanol–water solution (9:1 ratio) and stirred for 4 min. The mixture was then centrifuged (Thermo, Japan) at 3000 rpm for 5 min. Twenty microliters of the methanolic extract were mixed with 8.2 mL of water and 0.5 mL of Folin reagent. After 5 min, 1 mL of 10 % sodium carbonate was added, and the mixture was kept in the dark at room temperature for 1 h. The absorbance was measured at 765 nm using a UV–Vis spectrophotometer (Biochrom, UK). Gallic acid (0–1000 µg/mL) was used to create the standard curve, and total phenolic content was reported as mg of gallic acid per kg of sample [9].

### 2.7 Oxidative stability measurement

The optimal oil sample and control were tested for oxidative stability using a Rancimat device (Metrohm, Switzerland) at 110 °C, with an airflow rate of 20 L/h [17].

### 2.8 Fatty acid profile determination

After determining the best treatment, fatty acid methyl esters were prepared, and their profile was analyzed using gas chromatography (Agilent, USA) equipped with a 60 m × 0.25 µm film thickness silica capillary column. The initial temperature was set at 80 °C, increased by 15 °C/min to 200 °C, held for 10 min, and then increased to 220 °C for 5 min. The injector and detector temperatures were set at 210 °C, and helium was used as the carrier gas at a flow rate of 1 mL/min. The resulting curve was compared to a standard curve to quantifythe fatty acids in the oil, expressed as percentages [17].

### 2.9 Experimental design and statistical analysis

Response surface methodology (RSM) was applied using a central composite rotatable design to evaluate the effects of fixed parameters (ultrasound time and screw press speed) on variable parameters (oil extraction efficiency, acidity, peroxide value, refractive index, and total phenolic content). This design allowed estimation of all coefficients in the second-degree regression model and the interaction effects of factors. This research focused on studying the interaction effects and identifying the optimal conditions for extracting oil from camelina seeds using ultrasound pretreatment. For analyzing the response surfaces, a second-degree polynomial equation was fitted to each independent variable. The accuracy and fit of the regression model were evaluated using analysis parameters, lack of fit, and the coefficient of determination (R^2^). Statistical analysis was performed using Design-Expert software, version 12.

## 3 Results and discussion

### 3.1 Effect of studied parameters on oil extraction efficiency

**Fig. 1:**
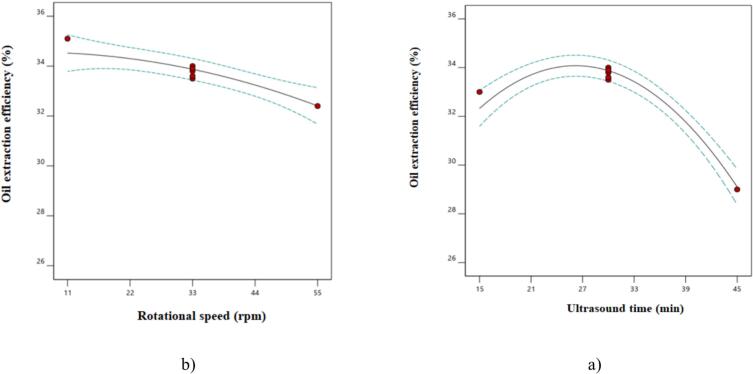
The effect a) ultrasound time and b) rotational speed on oil extraction efficiency from camellia seeds.

The statistical analysis (Table 1) indicates that the linear parameters of ultrasound time, screw press speed, and the second-degree parameter of ultrasound time had significant effects on oil extraction efficiency (p < 0.05). Fig. 1a shows that oil extraction efficiency initially increased with ultrasound time but then decreased. In contrast, the efficiency consistently decreased with higher screw press speeds (Fig. 1b).Ultrasound waves primarily cause the collapse and explosion of cavitation bubbles. As ultrasound time increases, cavitation bubbles form and collapse with greater intensity. The force generated by bubble explosions disrupts cell walls, enhancing mass transfer of oil from the seed to the solvent [9]. These findings are consistent with the results of Esposito and Piazza [18], who studied the effect of ultrasound waves on oil extraction from hemp. However, when the ultrasound time was extended further, the oil extraction efficiency likely decreased due to the possible destruction of oil exit pores [7].

**Table 1:** Analysis of variance for determined parameters in oil extraction by ultrasound pretreatment.

	Oil extraction efficiency		Oil Acidity		Oil Peroxide		Total Phenol		Refractive Index	
Source	Sum of squares	F Value	Sum of squares	F Value	Sum of squares	F Value	Sum of squares	F Value	Sum of squares	F Value
Model	58.67^**^	60.41	0.083^**^	16.68	0.55^**^	28.34	2451.44^**^	42.04	0.00	−
X_1_	15.52^**^	70.90	0.049^**^	19.67	0.25^**^	63.69	368.17^**^	31.57	−	−
X_2_	6.72^**^	34.60	0.034^**^	13.69	0.19^**^	49.91	640.67^**^	54.94	−	−
X_1_X_2_	0.46^ns^	2.35	−	−	0.048^**^	12.43	64.00^ns^	5.49	−	−
X_1_^2^	27.57^**^	14.94	−	−	0.06^**^	15.52	1141.25^**^	97.86	−	−
X_2_^2^	0.46^ns^	2.38	−	−	0.005^ns^	1.25	1.89^ns^	0.16	−	−
Residual	1.36	−	0.025	−	0.027	−	81.63	−	0.00	−
Lack of Fit	1.19 ^ns^	9.21	0.024 ^ns^	15.99	0.026 ^ns^	68.58	71.63	9.55 ^ns^	0.00	−
Pure Error	0.17	−	0.001	−	0.0005	−	10.00	−	0.00	−
Cor Total	60.03	−	0.108	−	0.579	−	2533.08	−	0.00	−

X_1_ and X_2_ are ultrasonic time and press speed, respectively and **, * and ns in each column indicate significance at level % 1, 5 and non-significance, respectively.

Additionally, higher screw press speeds reduced oil extraction efficiency due to lower pressure on the seeds, as the seeds exited the device faster [1]. Similar results were observed in studies by Saghali et al. [7] and Haji-Moradkhani et al. [19], where higher screw press speeds reduced oil extraction efficiency.

The equation for oil extraction efficiency from camelina seeds is shown in Table 2. Based on this model and the F-values in Table 1, the second-degree parameter of ultrasound time had the most significant effect on oil extraction efficiency.

**Table 2:** Designed equation models for dependent variable.

**NO**	**Properties**	**Equation**	**R^2^**	**R^2^-adj**	**CV**
1	Oil extraction efficiency	Y=+33.87–1.61X_1_– 1.06X_2_ – 3.16X_1_^2^ – 3.16X_2_^2^ – 0.41X_1_X_2_	0.977	0.961	4.67
2	Oil acidity	Y=+ 0.52 + 0.090X_1_ + 0.075X_2_	0.769	0.723	9.55
3	Oil peroxide	Y=+ 1.22 + 0.20X_1_ + 0.18X_2_ –0.15X_1_^2^ + 0.042X_2_^2^ + 0.11X_1_X_2_	0.53	0.919	5.34
4	Total phenol	Y=+ 113.38– 7.83X_1_– 10.33X_2_ –20.33X_1_^2^ – 0.83X_2_^2^ + 4.00X_1_X_2_	0.968	0.945	3.30
5	Refractive indexes	Y=+ 1.48	0.00	0.00	0.00

### 3.2 The effect of studied parameters on acidity levels

**Fig. 2:**
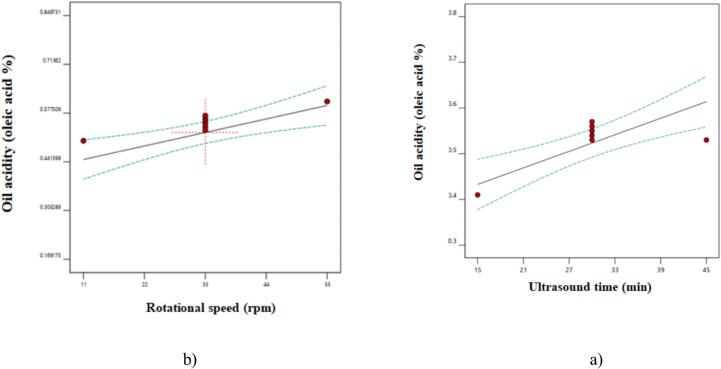
The effect a) ultrasound time and b) rotational speed on oil acidity.

Studies have shown that the oil extraction yield is the most important factor in evaluating methods of oil extraction from oilseeds. However, oxidative parameters, such as acidity, are also crucial when selecting an oil extraction method [11]. Table 1 indicates that only the linear parameters studied had a significant effect on oil acidity at the 5 % level. Other quadratic parameters and interaction effects were excluded from the final model because a linear model was selected for this test (Table 2). Fig. 2 shows that increasing sonication time and pressing speed led to higher oil acidity. The lowest acidity was observed at a sonication time of 15 min and a pressing speed of 11 rpm. This increase in acidity can be attributed to the greater activity of lipase enzymes, which results in the production of fatty acids in the oil [20]. Similarly, Thilakarathna et al. [11] demonstrated that sonication causes the partial breakdown of the cell membrane, leading to the release of more enzymes and, consequently, an increase in oil acidity. Esposito and Piazza [18] and Moghimi et al. [9] also reported that the use of ultrasound increases oil acidity. Additionally, Mohseni et al. [21] and Gharavi et al. [22] demonstrated that increasing the pressing speed raises the temperature, thereby increasing oil acidity. This aligns with the results of this study. Based on the coefficients of the fitted model for oil acidity, the linear parameter of sonication time had a greater impact on this characteristic than pressing speed.

### 3.3 The impact of studied parameters on peroxide levels

**Fig. 3:**
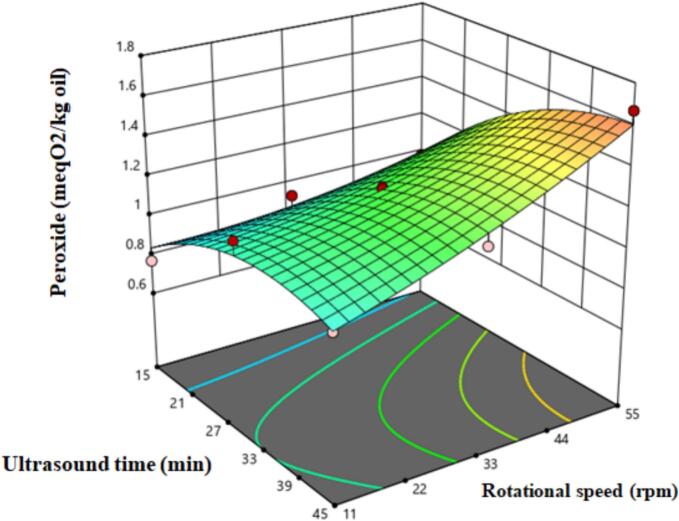
The effect ultrasound time and rotational speed on oil peroxide.

Since a higher degree of unsaturation in oils leads to a greater tendency for oxidation [1], this test was conducted. The results of the statistical analysis indicated that all linear parameters, their interactions, and the second-degree parameter of ultrasound time significantly affected the peroxide levels of the oil (Table 1). As shown in Fig. 3, increasing ultrasound time and rotational speed of the press led to higher peroxide levels in the samples. This increase can be attributed to greater oxidation of fatty acids, the production of free radicals due to high temperatures, and increased enzyme activity resulting from damage to the cell structure [23].

Studies by [24]and [23] also align with these results, showing that prolonged ultrasound exposure leads to increased peroxide levels in the oil. Similarly, Bakhshabadi et al. [1] demonstrated that increasing the rotational speed of the press during oil extraction from black cumin seeds resulted in higher peroxide levels in the oil. The equation for fitting the peroxide data is provided in Table 2. Based on this model and the F-values in Table 1, the linear parameter of ultrasound time had the most significant influence on peroxide levels.

### 3.4 The impact of studied parameters on total phenols

**Fig. 4:**
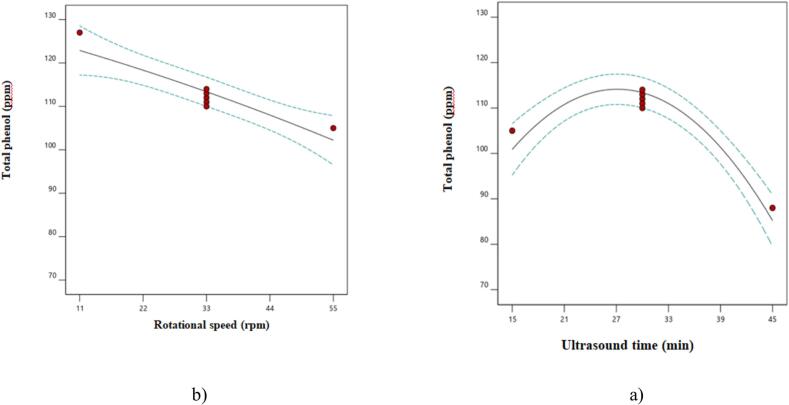
The effect a) ultrasound time and b) rotational speed on total phenol.

The data presented in Table 1 indicate thatall linear parameters and the second-degree parameter of ultrasound time significantly affected total phenols. In contrast, the second-degree parameter of press rotational speed and the interaction effects of the studied parameters were not significant at the 5 % level. As shown in Fig. 4, the total phenol content of the samples was initially enhanced by increasing the ultrasound time, but this was followed by a decrease. However, as the rotational speed of the press increased, the total phenol content of the oils consistently decreased. The increase in phenolic compounds can be attributed to their greater release caused by cell rupture from ultrasound waves [9]. In contrast, the decrease in these compounds is likely due to the destructive effects of prolonged ultrasound exposure. The findings of this section contrast with those of Rombaut et al. [25] and Bakhshabadi et al. [1], who reported that increasing the rotational speed of the press leads to higher total phenolic compounds. However, they align with the results of Saghali et al. [7]. The equation for fitting the total phenol data, presented in Table 2, suggests that the second-degree parameter of ultrasound time had a more significant effect on this characteristic.

### 3.5 The impact of studied parameters on the refractive index of oils

Since none of the examined models for the refractive index of oils were significant (p < 0.05), it can be concluded that the studied parameters had no effect on the refractive index of the oils (Table 1). The results showed that the refractive index of all samples was 1.4750. Different fats and oils have specific refractive indices, making this characteristic useful for identifying and determining the purity of oils and fats. The refractive index is also useful for monitoringthe progress of reactions such as hydrogenation and catalytic isomerization of oils. Additionally, the refractive index is used to detect oil oxidation, with temperature and unsaturation being key influencing factors [1,7]. Studies by Haji‐Moradkhani et al. [19] and Mohseni et al. [21], which focused on hemp seed and black seed, respectively, also demonstrated that different oil extraction processes have no significant impact on the refractive index.

### 3.6 Optimization of the oil extraction process from camelina and comparison of the oil obtained under optimal conditions with the control sample

To determine the optimal extraction conditions for oil from camelina seeds using ultrasound pretreatment, the ultrasonic treatment time was set between 15 and 45 min, and the screw press rotational speed was adjusted from 11 to 55 rpm. The oil extraction process under these conditions was optimized to maximize oil yield and total phenolic content, while minimizingacidity and peroxide levels. The results indicated that to achieve these objectives, the ultrasound time should be set at 21.02 min, and the screw press rotational speed should be 11 rpm. Under these optimal conditions, a desirability value of 0.862 was achieved. To compare the oil obtained under optimal conditions with a sample extracted without ultrasound pretreatment, a pressing speed of 11 rpm was used. The results showed that using ultrasound as a pretreatment for oil extraction increased the oil extraction yield, acidity, peroxide levels, and total phenolic content, but had no significant effect on the refractive index of the oils. Under optimal conditions, the use of ultrasound experimentally indicated a 20.76 % increase in oil compared to the method without ultrasound (Table 3). Additionally, a comparison of the optimization results with the experimental findings under the same conditions demonstrated the high accuracy of the predicted models. Moghimi et al. [9] reported that ultrasound improves the extraction of beneficial compounds from black seed. Their study investigated the effects of power and ultrasound time on the phenolic compounds and some physicochemical properties of cold-pressed black seed oil.

**Table 3:** Data obtained from optimal conditions, experimental data and control sample in this study.

Variables	Oil extraction efficiency (%)	Oil acidity (% oleic acid)	Peroxide (meqO_2_/kg oil)	Total phenol (ppm)	Refractive indexes
Optimal conditions	34.15	0.394	0.97	122.68	1.4750
Experimental data	34.90	0.395	0.95	123.64	1.4750
Control	28.90	0.310	0.65	73.00	1.4750

### 3.7 Evaluation of oxidative stability

Oxidative stability refers to the duration required to reach a point where one of the oxidative parameters (such as peroxide or carbonyl value) increases significantly after a steady rise, leading to the development of undesirable taste and odor in the oil [1]. The results showed that ultrasound pretreatment reduced oxidative stability from 3.75 h to 3.13 h (Table 4). This decrease in oxidative stability can be attributed to the reduction in tocopherol compounds caused byincreased heat [26,27]. Additionally, the higher acidity and peroxide levels in oils obtained from ultrasound pretreatment contributed to the reduction in stability. Bakhshabadi et al. [1] investigated the effects of microwave treatment and pressing speed on black seed oil and found that oxidative stability decreases with increasing process time and pressing speed. In this study, however, ultrasound reduced the oxidative stability of the oil by approximately 0.62 h.

**Table 4:** Oxidative stability of the studied samples.

Sample	Oxidative stability (h)
Control	3.75 + 0.05^a^
Optimal conditions	3.13 + 0.03^b^

Different lowercase letters in each column indicate significance at the 5% level.

### 3.8 Effect of ultrasound pretreatment on the fatty acid profile of camelina oil

The fatty acid composition of camelina oil, influenced by the optimal pretreatment and the untreated sample (control), is shown in Table 5. As observed, linolenic acid was the predominant fatty acid present in both samples. Additionally, ultrasound pretreatment increased the levels of saturated fatty acids, specifically palmitic and stearic acids, while decreasing the levels of certain unsaturated fatty acids (p < 0.05). Fallah et al. [28] demonstrated that linolenic acid is the dominant fatty acid in camelina oil, with significantly higher levels than those in soybean oil. The results regarding the impact of various pretreatments on the fatty acid composition indicated only minorchanges in some fatty acids. For instance, the findings of this study align with those of Saghali et al. [7], who reported that ultrasound waves caused negligible changes in certain fatty acids in sunflower oil.

**Table 5:** Fatty acid composition of camellia oil under the influence of different treatments.

Fatty acids	Structure	Control	Optimal conditions
Palmitic acid	C16	0.05 ± 0.001^i^	0.04 ± 0.001^h^
Stearic acid	C18	7.99 ± 0.002^c^	9.4 ± 0.020^c^
Oleic acid	C18:1(9)	0.08 ± 0.001^h^	0.07 ± 0.001^g^
Linoleic acid	C18:2(9,12)	5.6 ± 0.003^d^	5.6 ± 0.012^d^
Linolenic acid	C18:3(9,12, 15)	40 ± 0.500^b^	39.7 ± 0.360^b^
Arachidic acid	C20	43.7 ± 0.471^a^	43.3 ± 1.250^a^
Eicosenoic acid	C20:1	0.9 ± 0.056^e^	0.7 ± 0.042^e^
Eicosadienoic acid	C20:2	0.7 ± 0.003^f^	0.6 ± 0.061^e^
Behenic acid	C22	0.2 ± 0.001^g^	0.2 ± 0.001^f^
Erucic acid	C22:1	0.05 ± 0.001^i^	0.02 ± 0.001^i^

Different lowercase letters in each column indicate significance at level 5%.

## 4 Conclusion

The main objectives of this study were to enhance oil extraction from camelina seeds using ultrasound pretreatment and to optimize the oil extraction process. The overall results of this study demonstrated that ultrasound pretreatment increased both extraction yield and phenolic compounds in camelina oil, highlighting its positive effect on oil extraction. In this study, the oil extraction efficiency from camelina seeds was increased by 20.76 % using ultrasound. To achieve optimal conditions for oil extraction, an ultrasound time of 21.02 min and a screw press rotational speed of 11 rpm were necessary. However, due to the low oxidative stability and high linolenic acid content of this oil, it is advisable to mix it with other oils rather than used alone.

## CRediT authorship contribution statement

**Samira Mansuri:** Writing – original draft, Investigation. **Hamid Bakhshabadi:** Writing – review & editing, Supervision. **Masumeh Moghimi:** Validation, Methodology, Conceptualization. **Aminallah Tahmasebi:** Writing – review & editing, Software, Project administration. **Mehdi Gharekhani:** Methodology, Data curation.

## Declaration of competing interest

The authors declare that they have no known competing financial interests or personal relationships that could have appeared to influence the work reported in this paper.
